# Development of a new valid and reliable microsurgical skill assessment scale for ophthalmology residents

**DOI:** 10.1186/s12886-018-0736-z

**Published:** 2018-03-05

**Authors:** Zhihua Zhang, Minwen Zhou, Kun Liu, Bijun Zhu, Haiyun Liu, Xiaodong Sun, Xun Xu

**Affiliations:** 1 Shanghai Key Laboratory of Ocular Fundus Diseases, Shanghai, China; 2 Department of Ophthalmology, Shanghai General Hospital, Shanghai Jiao Tong University School of Medicine, 100 Haining Road, Shanghai, 200080 China; 3 Shanghai Engineering Center for Visual Science and Photomedicine, Shanghai, China

**Keywords:** Assessment scale, Cornea suturing, Medical education, Microsurgical skill

## Abstract

**Background:**

More and more concerns have been arisen about the ability of new medical graduates to meet the demands of today’s practice environment. In this study, we wanted to develop a valid, reliable and standardized assessment tool for evaluating the basic microsurgical skills of residents in a microsurgery laboratory, to get them well prepared before entering the surgical realm of ophthalmology.

**Methods:**

Twenty-three experts who have teaching experience reviewed the assessment scale. Constructive comments were incorporated to ensure face and content validity. Twenty-one attendings from different specialties then graded eight corneal rupture suturing videos with the scale to investigate interrater reliability. Fourteen of them graded the same videos 3 months later to investigate intrarater reliability (repeatability).

**Results:**

A total of 280 assessment scales were completed. All the ICC values of interrater reliability were greater than 0.8 with 75% data greater than 0.9 (range 0.860–0.976). All the ICC values of intrarater reliability (repeatability) were also greater than 0.8 with 63% data greater than 0.9 (range 0.833–0.954).

**Conclusions:**

The assessment scale we developed is valid and reliable. This tool could be useful to ensure that junior residents achieve a certain level of microsurgical technique in a laboratory environment before training in the operation room. Hopefully, this tool will provide a structured template for other residency programs to assess their residents for basic microsurgical skills.

## Background

Along with the development of ophthalmic medical education, the training of surgical skills has become a key part of it. More and more educators have realized the importance of residents’ competence in the operating room; however, the traditional methods for assessing surgical skills are largely subjective. Those methods were lack of standardization, consistency and reliability. Moreover, for the student assessed, they didn’t know the standards and goals of surgical training. In order to change the condition, educators worldwide had done a lot of work. A variety of surgical competency assessment tools had been developed by international ophthalmic educators, such as OASIS (Objective Assessment of Skills in Intraocular Surgery), GRASIS (Global Rating Assessment of Skills in Intraocular Surgery), OSACSS (Objective Structured Assessment of Cataract Surgical Skill) and OSCAR (Ophthalmology Surgical Competency Assessment Rubric), and the feedback from experts and application of those assessments showed excellent results [1–7]. By far, most of the assessments focus on the performance of residents during real-life operations, especially cataract surgeries.

China is a developing and industrialized country. Ocular rupture especially corneal rupture is a common and dangerous ophthalmic emergency, which usually is residents’ first independent real-life surgery. Prompt and meticulous wound management may reduce severe postoperative complications such as wound leak and endophthalmitis [8]. Thus, residents should be well prepared before they go into the operation room. What’s more, suturing technique is a critical and fundamental part of microsurgery. Standardized and adept micromanipulation and suturing would pave the way for entering the surgical realm of ophthalmology. Therefore, in Shanghai, suturing corneal rupture on pig eyes is mandated to be one of the periodical exams of residency program. Appropriate evaluation of this procedure is essential because weaknesses in training and teaching are difficult to correct without factual data [9, 10]. Since no rating assessment for suturing corneal rupture has been created before, Chinese ophthalmic education workers need to develop a comprehensive assessment scale in response to the current demand. In this study, we aimed to establish an efficient and reliable assessment scale for suturing corneal rupture to ensure the basic surgical competency of residents.

## Methods

**Fig. 1:**
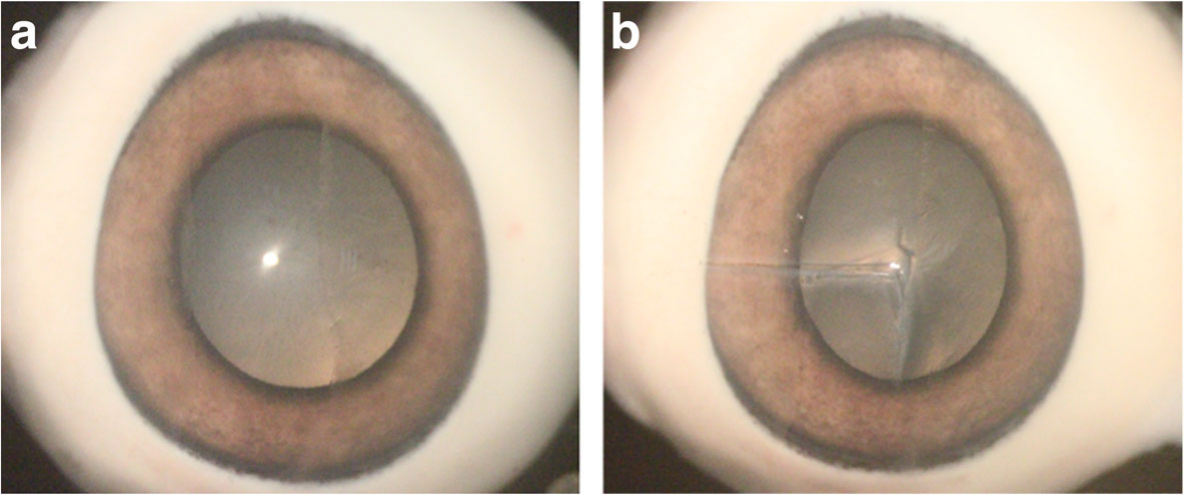
Illustrations of fresh pig eye for microscopic suturing in wet lab. **a**. Fresh pig eye before incision was made; **b**. “L” shaped incision was made on pig eye

This study was approved by the Ethics Committee of Shanghai General Hospital. All the operations were performed in a microsurgery laboratory using pig eyes (Fig. 1a). Each resident was given detailed information of what they were going to perform. The ruptures were “L” shaped involving the limbus. First, we made a full-thickness horizontal incision (about 6 mm) from 9 o’clock limbus to central cornea. The incision was then extended down for another 3 mm vertically (Fig. 1b). All necessary instruments, as well as distracter instruments, were laid out on the table. The whole process from gloves on to gloves off was videotaped and stored for later view. Senior attendings from different specialties were asked to watch those recorded videos and finish the assessment scales accordingly. The videotapes were chosen from residents at different rotating levels to include a range of surgical skills, and evaluators were blinded to the resident’s level of training. What’s more, 3 month later, each attending was asked to watch the same videos and complete the scales again. In order to avoid the recall of the last scoring, the playing order of the videos was changed.

### Validity of the assessment scale

**Fig. 2:**
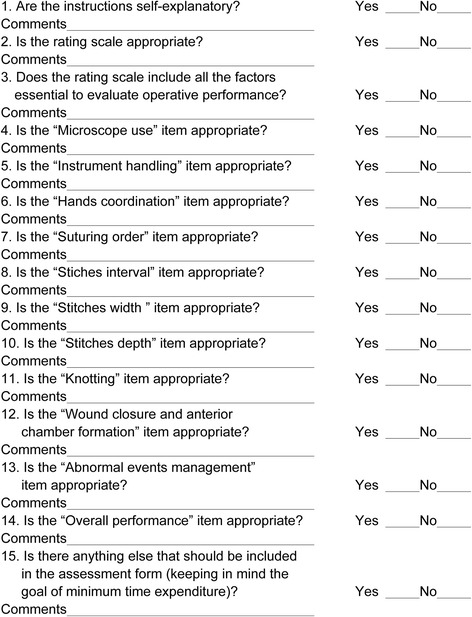
Survey sent to experts to determine the face and content validity of the assessment scale

A questionnaire was created (Fig. 2) to evaluate the scale’s face validity (i.e., the extent to which the components address the vital aspects) and content validity (i.e., the extent to which the components assess resident competency and skill) [3, 7]. The questionnaire along with the assessment scale was sent to experts from several teaching and research offices including one member of the committee of Shanghai standardized residency program, and then the scale was revised according to their comments and suggestions.

### Reliability and repeatability of the assessment scale

Senior attendings from different specialties were included in this evaluation to achieve a broad representation. The interrater reliability of different observers as well as the intrarater reliability of the same observer (repeatability) was tested using the intraclass correlation coefficient (ICC) [11]. The ICC is defined as the ratio of the between-subjects variance to the sum of the combined within-subjects and between-subjects variance [12]. ICC can very between 0 and 1, with 1 indicating perfect agreement. It should be greater than 0.7 in order for newly developed scales to be considered reliable [13–15]. We calculated the ICC using SPSS version 13.0 (Chicago, IL, USA). Considering the fact that we had a sample group of observers and cases, we used the Two-Way Random model. The Single Measures results were used to evaluate repeatability, and the Average Measures results were used for reliability. The significance level and confidence coefficients were set to 0.05 and 0.95, respectively.

## Results

### Validity of the assessment scale

Twenty-three experts completed the questionnaire, and the results of the questionnaire were noted in Table 1. Four experts recommended adding an assessment of “preoperative preparation and postoperative cleaning up” to the scale since the videotapes contained those parts and they were aspects of surgical skills. Two experts expressed that some of the descriptors were too explicit and burdensome to read and simplification may be better. Three experts suggested to use separated rating scales for “knotting”, “knots tightness”, and “knots exposure”. One expert commented to add “Suturing” to the scale to assess the general suturing performance of the students such as needle load and needle entry. Five experts felt there was no need to include an assessment of “abnormal events management”. All comments and suggestions were considered, and appropriate suggestions were incorporated into the assessment scale, thus establishing a level of face and content validity [6].

**Table 1:** Results of the Content and Face Validity Survey

Are those items appropriate?	Percentage
Microscope use	21/23 (91%)
Instrument handling	21/23 (91%)
Hand coordination	23/23 (100%)
Suturing order	22/23 (96%)
Suturing interval	23/23 (100%)
Suturing width	23/23 (100%)
Suturing depth	23/23(100%)
Knotting	20/23 (87%)
Wound closure and anterior chamber formation	23/23 (100%)
Abnormal events management	18/23 (78%)
Overall performance	23/23(100%)

Reported as the fraction (percent) of respondents answering “Yes” to the question

The finalized assessment scale was shown in Table 2. This assessment scale includes 6 measures of basic surgical skills (preoperative preparation, microscope use, instrument handling, hands coordination, postoperative clean up and overall performance) and 9 measures of the stages of suturing (suturing, suturing order, sutures interval, sutures width, sutures depth, knotting, knots tightness, knots exposure and wound leakage and anterior chamber formation), which are rated on a 5-point Likert scale, with each point anchored by explicit behavioral descriptors.

**Table 2:** Assessment Scale of Corneal Rupture Suturing

DATE _____ RESIDENT _____ EVALUATOR _____	1	2	3	4	5	Score
Preoperative preparation	Failed to wear hat, mask and gloves	Failed to wear two of the three	Failed to wear one of the three	Wearing hat, mask and gloves correctly	Wearing hat, mask and gloves smoothly	
Microscope use	Out of center and focus constantly	Out of center and focus frequently	Out of center and focus occasionally	Stay in center and focus constantly	Fluid moves with microscope	
Instrument handling	Constantly makes tentative and awkward moves with instruments by impropriate use	Frequently makes tentative and awkward moves with instruments	Fair use of instruments but occasionally stiff or awkward	Competent use of instruments	Fluid moves with instruments	
Hands coordination	Severely hands tremor and constantly instruments collision	Hands tremor and frequently instruments collision	Mild hands tremor and occasionally instrument collision	No hands tremor and instrument collision	Steady hands and perfect hands coordination	
Suturing	Sutures are done in an awkward, slow fashion with much difficulty. Bent needles	Sutures are done with difficulty	Sutures are done with little difficulty	Sutures are done properly. Loads needle 1/2 to 2/3 from tip. Approaches eye with flat portion of needle. Needle enters perpendicular to cornea	Smooth and perfect suturing. Always loads needle 1/2 to 2/3 from tip. Always approaches eye with flat portion of needle. Needle enters perpendicular to cornea	
Suturing order	Suture the rupture randomly	Suture the rupture in one direction	Selectively suture the rupture. Close the center first	Selectively suture the rupture. Close the angle first	Selectively suture the rupture. Surgical exploration of the limbus. Close the limbus first, then the angle	
Stitches interval	Awfully uneven	Uneven	Almost even	Even	Perfectly even, around 2 mm	
Stitches width	Awfully uneven	Uneven	Almost even	Even	Perfectly even, around 2 mm	
Stitches depth	Awfully uneven	Uneven	Almost even	Even	Perfectly even, around 2/3 of the cornea thickness	
Knotting	Knots are placed in an awkward, slow fashion with much difficulty	Knots are placed with difficulty	Knots are placed with little difficulty	Knots are placed properly with seldom breaking sutures	Knots are placed perfectly with no breaking sutures	
Knots tightness	Suture tightness is awfully uneven. Sutures are too tight or loose	Suture tightness is uneven. Sutures are tight or loose	Suture tightness is almost even. Sutures are a little bit tight or loose	Suture tightness is proper and even	Suture tightness is perfectly even. Sutures are placed tight enough to maintain the wound closed, but not too tight as to induce astigmatism	
Knots rotation	No suture rotation at all	Most of the sutures are not rotated	Parts of the sutures are not rotated	Most of the sutures are rotated	Complete suture rotation. No knots exposure	
Wound closure and anterior chamber formation	No wound closure and no anterior chamber formation	Part of wound closure and no anterior chamber formation	Questionable wound closure and anterior chamber formation	Complete wound closure and anterior chamber formation	Neat and watertight wound closure. Perfect anterior chamber formation with no anterior synechia of iris	
Postoperative clean up	Failed to clean up the pig eyes. Failed to settle the microscope and instruments. Failed to take off the hat, mask and gloves properly	Failed to do two of the three things	Failed to do one of the three things	Complete all the three things	Throw the pig eye in the yellow bag. Settle the microscope and instruments. Take off the hat, mask and gloves correctly	
Overall performance	Unable to finish the operation independently	Hesitant, frequent starts and stops. Finish the operation with difficulty	Occasional starts and stops. Finish the operation within 20mins	Competent, finish the operation within 15mins	Confident and fluid, finish the operation within 10mins	

### Reliability and repeatability of the assessment scale

Twenty-one attendings from different specialties finished 8-videotaped corneal suturing surgeries and completed the assessment scales accordingly for the first time. Specialties represented were cataract (4), glaucoma (3), cornea (3), strabismus (1), and retina (10). Only 14 attendings finished the scale again 3 month later. A total of 280 assessment scales were completed. All experts expressed that they could complete the scale within 5 min.

The interrater reliability of each surgical procedure step and overall score, considering 21 observers together, was summarized in Table 3. All the ICC values were greater than 0.8 with 75% data greater than 0.9. “Microscope use” Showed the highest reliability (0.976, 95%CI 0.942–0.994). The intrarater reliability (repeatability) of each step and overall score was listed in Table 4. All data were greater than 0.8, with 63% data greater than 0.9. “Suturing order” showed the highest repeatability (0.954, 95%CI 0.934–0.968).

**Table 3:** Interrater reliability of 23 observers for corneal rupture suturing assessing scale

	ICC	95% CI	
		Lower bound	Upper bound
Preoperative preparation	0.953^***^	0.888	0.989
Microscope use	0.976^***^	0.942	0.994
Instrument handling	0.940^***^	0.857	0.986
Hand coordination	0.963^***^	0.913	0.991
Suturing	0.866^***^	0.682	0.968
Suturing order	0.971^***^	0.932	0.993
Suturing interval	0.943^***^	0.863	0.986
Suturing width	0.939^***^	0.855	0.985
Suturing depth	0.860^***^	0.668	0.967
Knotting	0.922^***^	0.815	0.981
Knots tightness	0.886^***^	0.728	0.973
Knots rotation	0.913^***^	0.793	0.979
Wound closure and anterior chamber formation	0.892^***^	0.744	0.974
Postoperative clean up	0.920^***^	0.809	0.981
Overall performance	0.965^***^	0.917	0.992
Total score	0.959^***^	0.901	0.990

*ICC* intraclass correlation coefficient, *CI* confidential interval

***: *P* < 0.001

**Table 4:** Intrarater reliability (repeatability) for corneal rupture suturing assessing scale

Item	ICC	95% CI	
		Lower bound	Upper bound
Preoperative preparation	0.907^***^	0.867	0.935
Microscope use	0.934^***^	0.906	0.954
Instrument handling	0.866^***^	0.811	0.906
Hand coordination	0.904^***^	0.863	0.933
Suturing	0.865^***^	0.810	0.905
Suturing order	0.954^***^	0.934	0.968
Suturing interval	0.919^***^	0.884	0.943
Suturing width	0.901^***^	0.860	0.931
Suturing depth	0.885^***^	0.837	0.920
Knotting	0.916^***^	0.880	0.941
Knots tightness	0.833^***^	0.767	0.822
Knots rotation	0.843^***^	0.779	0.889
Wound closure and anterior chamber formation	0.901^***^	0.859	0.931
Postoperative clean up	0.893^***^	0.848	0.925
Overall performance	0.940^***^	0.915	0.959
Total score	0.946^***^	0.922	0.962

*ICC* intraclass correlation coefficient, *CI* confidential interval

***: *P* < 0.001

## Discussion

Investigations suggested a trend towards enhanced acquisition of microsurgical skill in students allowed to practice microsurgery on all kinds of simulators and/or in the wet laboratory [16–18]. Nevertheless, in the early twenty-first century, the ophthalmic education of residents in China was unstructured and of variable quality. There were more and more concerns arising about the ability of new medical graduates to meet the demands of today’s practice environment. Thus, China started the residency program about 10 years ago and Shanghai was one of the pilot cities. Up to now, each city is still responsible for its own resident training and examination. In Shanghai, the committee of ophthalmic resident training standardized the program as 3 years of ophthalmology education, and every year they will attend an annual ophthalmology residency-in-training examination. The major purpose of those examinations is to evaluate residents’ competence in 4 aspects: (1) medical knowledge, (2) patient care and communication skills, (3) case-based learning and analyzing, and (4) surgical skills. Suturing technique is a critical and fundamental part of microsurgery. Standardized and adept micromanipulation and suturing would pave the way for entering the surgical realm of ophthalmology. Therefore, the surgical skills of junior residents are assessed by performance on suturing corneal rupture on pig eyes. This kind of examination has been held for 5 years and the ophthalmic educators found out that the traditional scoring method might be unreliable due to grade inflation and overt subjective assessments [10, 19, 20]. Residency examination is supposed to enable competence in all aspects by collecting performance data that reliably and accurately reflects the resident’s real ability. Thus, a valid and reliable assessment tool is desperately needed.

To our knowledge, this is the first throughout assessment scale for corneal rupture suturing in wet laboratory. Fisher et al. [1] developed a phacoemulsification/wound construction and suturing technique assessment scale for ophthalmology residents, but suturing technique assessment was only part of the scale containing 8 general items. The scale was simple and only had 2 choices (not done/incorrect and done correctly). There was no behavioral or skill-based rubric for the observers to use when assessing the resident’s performance. Feldman et al. [21] used a corneal laceration repair assessment to evaluate microsurgical skill improvement after training on the simulator. However, the assessment was totally objective and only measured suture depth, bite size and suture spacing. In this study, we created a comprehensive, globally applicable assessment scale to evaluate the key components of corneal rupture suturing. This assessment scale breaks down to 15 essential items including 6 measures of basic surgical skills and 9 measures of the stages of suturing, with basic skill measures similar to that employed in GRASIS and OSCAR. Moreover, the scale is rated on a 5-point Likert scale with behavioral anchors for each level in each step of the surgical procedure.

The reliability and repeatability of the assessment tools mentioned above were seldom detected. In this study, we investigated validity, reliability and repeatability of our assessment scale. For validity, we asked 23 experts from different teaching and research offices, and all the comments were considered and appropriate suggestions were incorporated into the assessment scale. Therefore, a level of face and content validity was established. Considering the reliability for the entire group of 21 observers, the ICC values were higher than 0.8 (range 0.860–0.976) in all 15 individual categories as well as the overall score, indicating reliability of the tool as a whole. What’s more, the assessment scale yielded very good repeatability, with ICC values ranging from 0.833 to 0.954. An assessment scale is considered to give almost perfect outcomes when ICC value is 0.75 and above [13, 15, 22].

Drawbacks of the assessment scale are that it is relatively simple and it cannot provide information about resident’s judgment and handling of complications on real operations. However, it is a standardized tool that can be used to determine whether a resident is adequately prepared, in terms of their basic microsurgical skills, to enter the operating room. The “passing” threshold could be set at a score of > 3 for each item on the 5-point Likert scale. In addition, process in the wet laboratory can be standardized so that each resident is assessed under comparable circumstances, and ophthalmic educators can easily track their improvements or adjust the complexity to train residents of different rotating levels by changing the rupture (straight/ “Y” shaped rupture, with/without limbus).

## Conclusions

In this study, we aimed to create a standardized tool to assess basic surgical skills and to improve overall process of early surgical education. In summary, the assessment scale we developed is valid and reliable. It is an analytical scoring system that contains observable and measurable components of surgical performance. It will help educators to reduce the subjectivity of the assessment and clearly express to the residents what is expected to obtain competence. Hopefully, this tool will provide a structured template for other residency programs to assess their residents for basic surgical skills.

## Abbreviations

GRASIS: Global rating assessment of skills in intraocular surgery
ICC: Intraclass correlation coefficient
OASIS: Objective assessment of skills in intraocular surgery
OSACSS: Objective structured assessment of cataract surgical skill
OSCAR: Ophthalmology surgical competency assessment rubric
